# A MusD Retrotransposon Insertion in the Mouse *Slc6a5* Gene Causes Alterations in Neuromuscular Junction Maturation and Behavioral Phenotypes

**DOI:** 10.1371/journal.pone.0030217

**Published:** 2012-01-17

**Authors:** Laurent P. Bogdanik, Harold D. Chapman, Kathy E. Miers, David V. Serreze, Robert W. Burgess

**Affiliations:** The Jackson Laboratory, Bar Harbor, Maine, United States of America; Columbia University, United States of America

## Abstract

Glycine is the major inhibitory neurotransmitter in the spinal cord and some brain regions. The presynaptic glycine transporter, GlyT2, is required for sustained glycinergic transmission through presynaptic reuptake and recycling of glycine. Mutations in *SLC6A5*, encoding GlyT2, cause hereditary hyperekplexia in humans, and similar phenotypes in knock-out mice, and variants are associated with schizophrenia. We identified a spontaneous mutation in mouse *Slc6a5*, caused by a MusD retrotransposon insertion. The GlyT2 protein is undetectable in homozygous mutants, indicating a null allele. Homozygous mutant mice are normal at birth, but develop handling-induced spasms at five days of age, and only survive for two weeks, but allow the study of early activity-regulated developmental processes. At the neuromuscular junction, synapse elimination and the switch from embryonic to adult acetylcholine receptor subunits are hastened, consistent with a presumed increase in motor neuron activity, and transcription of acetylcholine receptors is elevated. Heterozygous mice, which show no reduction in lifespan but nonetheless have reduced levels of GlyT2, have a normal thermal sensitivity with the hot-plate test, but differences in repetitive grooming and decreased sleep time with home-cage monitoring. Open-field and elevated plus-maze tests did not detect anxiety-like behaviors; however, the latter showed a hyperactivity phenotype. Importantly, grooming and hyperactivity are observed in mouse schizophrenia models. Thus, mutations in *Slc6a5* show changes in neuromuscular junction development as homozygotes, and behavioral phenotypes as heterozygotes, indicating their usefulness for studies related to glycinergic dysfunction.

## Introduction

In the mammalian nervous system, inhibitory synaptic transmission is primarily mediated by vesicular release of gamma-aminobutyric acid (GABA) and glycine. GABAergic neurons predominate in the forebrain, while glycinergic neurons are primarily found in the brain stem and the spinal cord. However, inhibitory synaptic transmission is complicated, including examples such as the co-expression of GABA and glycine, and the role of glycine in particular needs further elucidation to determine its full impact in inhibitory circuitry, its role in neurological diseases, and its potential as a target for therapy.

In the spinal cord, motoneurons receive inhibitory connections from Renshaw cells, propriospinal interneurons, and brain stem projections [1], [2]. Renshaw cells are activated by axon collaterals of motoneurons innervating proximal muscles, and form a feedback loop onto the activating motoneuron or synergistic motoneurons (recurrent inhibition) [3]–[6]. Propriospinal interneurons relay inputs from the muscles or joints, and can connect directly, or via other spinal interneurons, to the motoneurons. Descending inputs from the ventromedial medulla also contribute GABA/glycinergic inhibition on the motoneurons [7]. Therefore, direct or indirect glycinergic inhibition onto spinal motoneurons modulates their excitability, and consequently, voluntary movements, central pattern generators responsible for locomotion and breathing, the strength of muscle contractions, and muscle relaxation.

In the sensory circuits of the dorsal horn of the spinal cord, glycinergic inhibition, often in association with GABAergic inhibition [6], [8], regulates the sensitivity to painful stimuli [9]. Glycinergic interneurons in different laminae of the dorsal spinal cord inhibit the transmission of low intensity stimuli to the brain. Pharmacological disinhibition of the spinal cord increases pain sensitivity (hyperalgesia) [10], [11], and modification of the glycinergic transmission in cases such as neuropathic pain, makes innocuous stimuli painful (allodynia) [12].

In the brain, most synaptic inhibition is mediated by GABA, but glycinergic terminals are found in low abundance in the thalamus, hypothalamus, cortex and cerebellum and in high abundance in the brain stem and cerebellum [13]. The role of glycinergic transmission in the brain is less understood, and is complicated by the fact that glycine can be co-released with GABA [14], can be released by astrocytes [15] and can diffuse (spill-over) from inhibitory synapses and potentiate NMDA receptors [16], [17].

Glycine is released from presynaptic terminals through vesicular mechanisms, and classically acts on its postsynaptic receptors, which are ligand-gated pentameric chloride channels [18]. Glycine is cleared from the synaptic cleft by two glycine transporters, GlyT1 (Solute carrier, family 6, member 9, *Slc6a9*) found primarily on astrocytes, and GlyT2 (Solute carrier, family 6, member 5, *Slc6a5*), found primarily on presynaptic terminals [19]. GlyT1 terminates glycinergic transmission by clearing glycine from the synapse, whereas GlyT2 is needed to sustain prolonged glycinergic transmission through presynaptic reuptake and vesicle reloading in the inhibitory presynaptic terminal [20]. The localization of GlyT2 to glycinergic terminals [13] makes it a reliable marker of glycinergic neurons and a target of choice to specifically alter glycinergic synaptic transmission.

Genetic experiments in mice and elucidation of congenital disorders in humans have confirmed and refined the understanding of glycinergic transmission gained by histological and pharmacological approaches. Mice lacking *Slc6a9* expression display an absence of muscle tone and irregular breathing. On in vitro preparations of the brain stem, containing the respiratory network, *Slc6a9* mutants display prolonged inactivity instead of the repetitive firing pattern observed in wild-type animals, and this defect is reversed by the GlyR blocker strychnine. These observations together indicate that loss of GlyT1 leads to an excessive spinal glycinergic inhibition, and prove the requirement of GlyT1 in the clearance of glycine from the extracellular milieu [21]. Alterations of *SLC6A9* are suspected to cause glycinergic encephalopathy in humans, although no mutation in patients has been detected to date [22]. In contrast, mice lacking *Slc6a5* expression have muscle tremors and generalized spasms [23] and recordings from the spinal cord motoneurons revealed a strong reduction of the miniature inhibitory currents amplitude, indicating a lack of glycinergic inhibition, consistent with the requirement of GlyT2 for the reuptake and recycling of glycine at the presynaptic terminal. In humans, *SLC6A5* mutations cause a similar phenotype of perinatal hyperekplexia, a condition characterized by stiff muscles [24], [25]. Mutation or deletion in *Slc6a5* also causes postnatal handling- or noise-induced myoclonus in calves [26] and muscle stiffness and tremor in canine puppies [27], phenotypes very similar to those observed in mice. Similarly, defects in another component of the glycinergic pathway, gephyrin, cause loss of glycine receptors from the synapse and rigidity of neonatal mice, consistent with an absence of glycinergic inhibition [28] and stiff man syndrome and related disorders in humans [29], [30]. Surprisingly, while mutations in *Slc6a5* are lethal postnataly in mice, dogs and cows, they only cause a transient, postnatal muscle stiffness in humans that disappears in the first year and only reappears with lower severity in conjunction with an increased startle response in adults with hyperekplexia. The lethality in other organisms appears to result from the extreme spasticity in response to voluntary movement, which eventually prohibits even basic behaviors, whereas with care, humans can eventually compensate with increased GABAergic inhibition.

In regard to modulation of pain sensitivity by glycine, the alpha3 glycine receptor was found to specifically mediate spinal cord processing of nociception and prostaglandin-mediated pain sensitization [31] and mice lacking alpha3 were immune to inflammatory pain sensitization. Consistently, activation of alpha3 GlyRs has also been shown to mediate the cannabis analgesic effects independent of the cannabinoid receptors [32].

In addition to the anticipated effects on muscle tone resulting from changes in glycinergic transmission in the spinal cord, glycine is increasingly implicated in higher behavioral functions and neuropsychiatric disorders. Local brain delivery of strychnine, a glycine receptor blocker, has been shown to be anxiolytic in rats [33]. Interestingly, adult mice heterozygote for the *Slc6a9* knock-out or with a targeted deletion of *Slc6a9* in the forebrain neurons, are free of any muscle tremor, but display an increase in spatial memory, a decrease in the sensitivity to amphetamine in the disruption of prepulse inhibition [34], better Pavlovian aversive conditioning, and better learning abilities in a special paradigm [35]. To date, no cognitive phenotype has been associated with *Slc6a5* in mice ; in humans, although first studies found no association between *SLC6A5* variations and schizophrenia [36], SNPs in the *SLC6A5* gene have been associated with schizophrenia in the Japanese population [37]. Some of these phenotypes may not be related to changes in inhibition, but instead to the role of glycine in potentiating NMDA receptors. For instance, a partial agonist of the glycine-binding site on the NMDA receptor, D-cycloserine, has also been shown to be mildly anxiolytic and a stimulant [38]. Therefore, additional behavioral testing in animal models that perturb glycinergic transmission is warranted.

In this study, we have found a new, spontaneous loss-of-function allele of *Slc6a5* in mice. The overt phenotype of handling induced muscle rigidity is similar to the previously described phenotype in the knockout strain. We were able to use this mutation as a homozygote to study the impact of changes in motoneuron excitability in the developmental processes of synapse elimination and maturation at the neuromuscular junction. We were also able to study the mutation as a heterozygote to determine the effects of reduced GlyT2 levels on sensory function and behavior. We conclude that glycinergic pathways, and GlyT2 in particular, are candidate targets for therapeutic interventions for multiple behavioral phenotypes related to neuropsychiatric conditions.

## Results

### Mapping and characterization of a new *Slc6a5* mouse mutant

**Figure 1:**
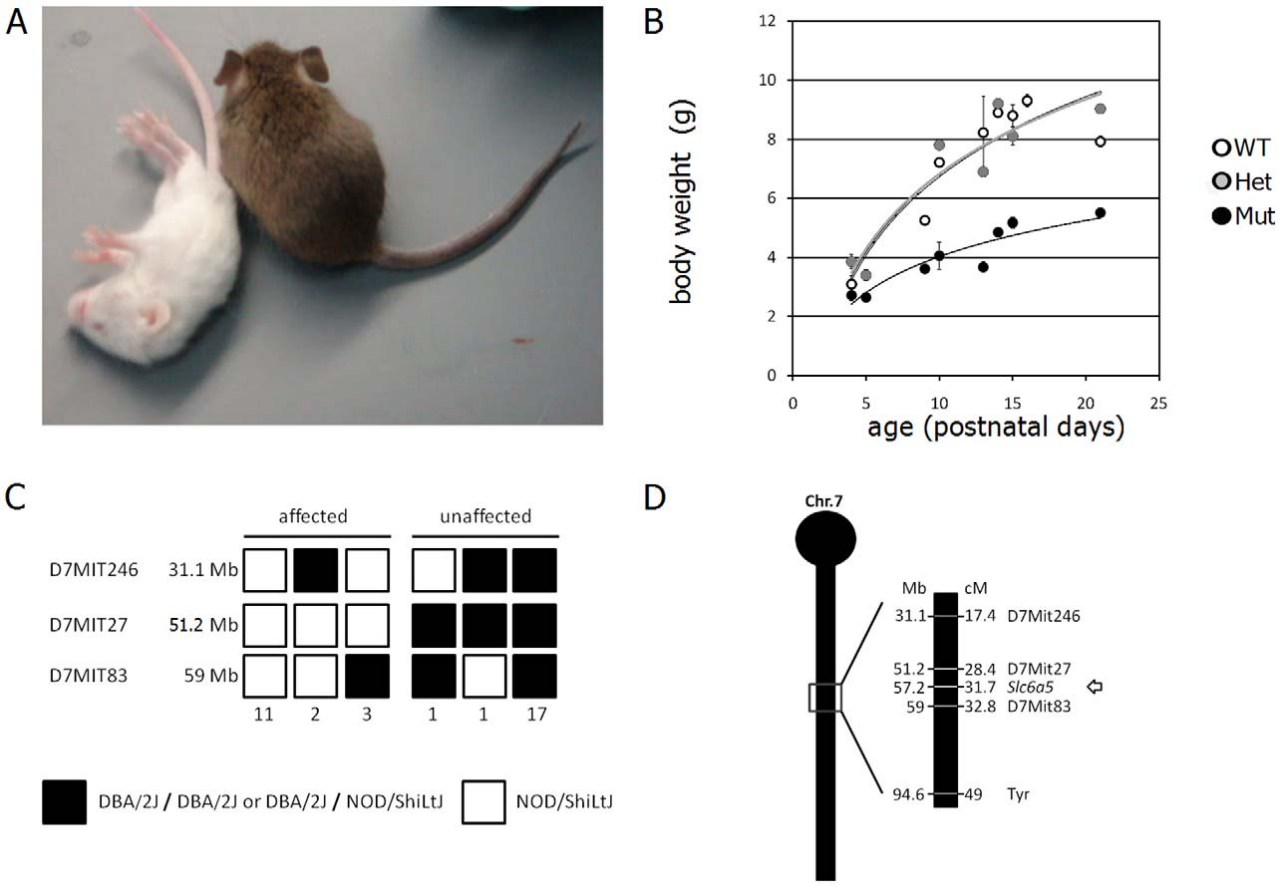
Overt phenotype and mapping of the new mutation. **A.** P15 mutant (left, white) and unaffected littermate (right, brown) in the NOD x DBA/2 mapping cross. Picture was taken at the time of a generalized tremor of the mutant. **B.** Growth curve for wild-type, heterozygous and homozygous mutants, completed once the genotyping assay was established. **C.** Haplotype of affected (left) and unaffected (right) mice at three loci of chromosome 7 flanking the *Slc6a5 (Slc6a5)* gene. Simple sequence length polymorphism (*Mit* markers) different in NOD and DBA/2 and their positions in megabases (Mb) are indicated. The number of mice of each genotype is indicated below the diagram. **D.** Schematic representation of chromosome 7 with centromere at the top, location of MIT markers used and *Slc6a5*. Positions are given in megabases (Mb) on the left and centimorgans (cM) on the right.

Mice with a putative spontaneous mutation were noted in an inbred NOD.129S2(B6)-Cd8a^tm1Mak^/DvsJ colony maintained at The Jackson Laboratory. This strain is typically used as a control strain for type 1 diabetes research, it does not develop a diabetic phenotype and does not have overt neuromuscular phenotypes. However, a subset of these mice displayed generalized muscle spasms and stiffness, particularly in response to handling (Fig. 1.A and Movie S1) around 5 days after birth, lower body weight first noticeable at P5 (Fig. 1.B), and survived only two weeks postnatally. Affected mice repeatedly arose in the litters obtained from unaffected parents at approximately one in four, suggesting a heritable defect caused by a single recessive locus. We therefore undertook a standard F2 mapping cross with DBA/2J as the wild type mapping partner (see methods). In the F2 progeny of this cross, the stiffness phenotype partially co-segregated with the white coat color caused by the recessive *albino* mutation of the *tyrosinase* (*Tyr*) gene in the NOD strain (the DBA/2J mice are Agouti), at 94.6 Mb on Chromosome 7. Of 95 F2 pups, 34% were affected, and 69% of the affected pups were albino, whereas only 8% of the non-affected pups were albino. The association with Chr. 7 was further confirmed by standard genetic mapping with simple sequence length polymorphism (*Mit*) markers using 16 affected and 19 unaffected littermate mice. This further localized the mutation between 31.1 and 59 Mb on chromosome 7, segregating with *D7Mit27* at 51.2 Mb in all mice examined (Fig. 1.C). Of all the genes documented by the Mouse Genome Informatics website in this region, *Slc6a5*, encoding GlyT2, at 57.2 Mb on the Chromosome 7 (Fig. 1.D), was selected for closer analysis because of the similarity of the phenotypes of the new mutant and the published *Slc6a5* knockout allele [23].

**Figure 2:**
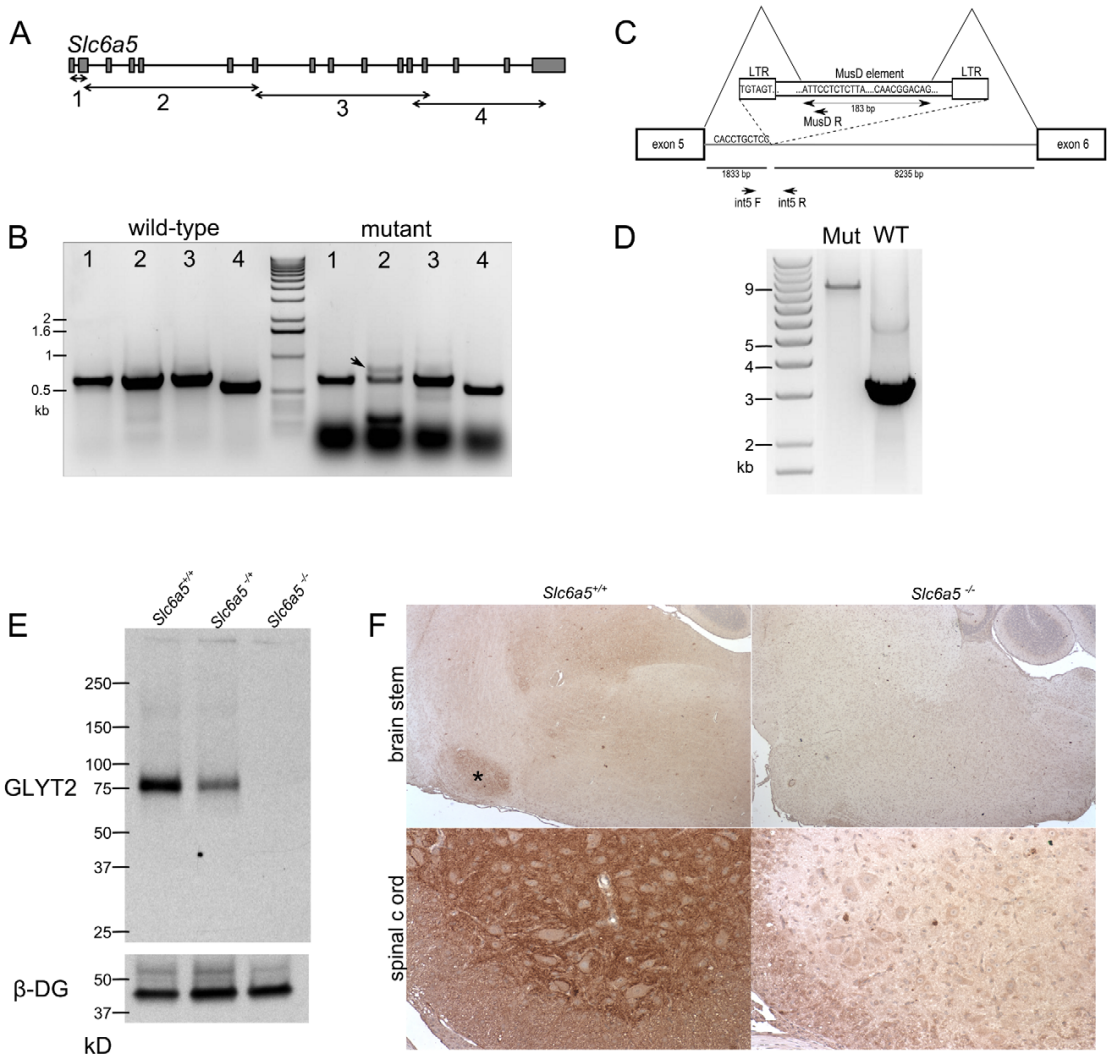
Characterization of the new *Slc6a5* mutation as a null-allele. **A.** Schematic of the *Slc6a5* gene organization, with boxes and lines representing exons and introns respectively, and boundaries of PCRs (1 to 4) run on reverse-transcribed cDNAs. **B.** Agarose gel of the PCR amplification products from cDNA. Note the extra band in PCR 2 on mutant brain cDNAs (arrow). **C.** Schematic representation of intron 5, and location of the insertion and splice donor and acceptor sites in the retrotransposon sequence. The 10 base pairs immediately 5′ of the insertion site, 1833 bp into intron 5, are shown, as well as the first base-pairs of the LTR element of the retrotransposon. The 183 bp sequence of the transposon that is spliced-in *Slc6a5* mRNA is indicated with its 10 first and last base-pairs and the inferred acceptor and donor splice sites. The primers used to localize the insertion (int5F and MusR), and to amplify the transposon from mutant genomic DNA (int5F and int5R) are indicated by arrows. **D.** Agarose gel of the products of the PCR on genomic DNA with primers flanking the insertion site (int5F/R of C.). A 3 kb product was amplified from wild-type gDNA, but only a 9 kb product was amplified from mutant gDNA. **E.** Anti-GLYT2 Western blot on spinal cord cell membrane preparations, with beta-dystroglycan used as a loading control. Genotypes are indicated above the blot. **F.** Anti-GLYT2 immunocytochemistry (DAB) on brain sagittal sections and spinal cord cross sections. Nuclei were counterstained with hematoxylin before mounting. The lateral brain stem is shown in the top panels with the ventral lobules of the cerebellum visible in the top right corners and the ventral horn of the spinal cord in shown in the bottom panels.

Possible variation in the *Slc6a5* gene was examined by non-quantitative RT-PCR from mutant and control brain mRNAs. Partially overlapping reactions were designed to amplify cDNA sequences encoded by exons 1 to 2, exons 2 to 7, exons 7 to 13 and exons 12 to 16 (Fig. 2.A). The reaction amplifying exons 2 to 7 produced two bands of near normal size and a third, very small band in the mutant mice presumed to be homozygous, but only one band in the wild-type (Fig. 2.B). When sequenced, the product similar in size to the wild type product was indeed indistinguishable from the reference sequence of C57BL/6J mice. However, the longer product included an insertion of 183 bp between exons 5 and 6 (Fig. 2.C). Using a BLAST search, these 183 bp were found to be perfectly homologous to the MusD LTR-retrotransposon [39](Fig. 1.C). A reverse primer in the 5′ region of the 183 bp retrotransposon insert (primer MusD R in Fig. 2.C) and forward primers in intron 5 of *Slc6a5* were used for amplification from genomic DNA of homozygote mutants. One of the forward primers (int5 F, Fig. 2.C) produced an amplification product. Sequencing showed that the retrotransposon inserted in the sequence of intron 5 at 1,833 base pairs. PCR on genomic DNA using primers flanking this site (int5 F and int5 R, Fig. 2.C), amplified a anticipated, 3 kb product from control mice, but a 9.5 kb product was amplified from homozygous mutant mice (Fig. 2.D). Based on sequence from the 5′ end of the 9.5 kb product, the difference in size was accounted for by an almost complete MusD retrotransposon. Taken together, these data indicate that the retrotransposon inserted in intron 5 contains splice donor and acceptor sites, resulting in a 183 bp fragment of the transposon being spliced into the mRNA of *Slc6a5* between exons 5 and 6 (Fig. 1.C). The insertion does not modify the splicing of the neighboring exons 2, 3, 4 and 7 (Fig. 1.B).

The effects of the MusD insertion were unclear since the 183 bp insertion preserves the reading frame of *Slc6a5*, wild type cDNA without the insertion was still detected (Fig. 2.B), and the RT-PCR used to find the mutation was not quantitative. Therefore, we analyzed *Slc6a5* mRNA and protein levels in the mutant mice. Quantitative RT-PCR on spinal cord cDNAs with primers designed on the 3′ end of the *Slc6a5* cDNA, downstream of exon 5, showed a reduced amount of transcript in the heterozygotes and a virtually undetectable level in the homozygote mutants (data not shown). By western blot, GlyT2 was detected at very low levels in the brains of wild-type animals (data not shown) but was readily detectable in spinal cord extracts (Fig. 2.E). In the mutant mice, no protein was detected with the antibody recognizing the C-terminal part of the protein, suggesting that the intronic insertion either interferes with the translation of *Slc6a5* or destabilizes *Slc6a5* transcripts and creates a null mutation. Importantly, in mice heterozygous for the mutation, the amount of GlyT2 protein was reduced by approximately 50% compared to wild-type samples. Using immunocytochemistry, GlyT2 was detectable in sagittal brain sections of wild type mice in the olive nuclei of the brainstem and the spinal cord, consistently with previous reports [40]–[41]. Brains and spinal cords from mutant mice were devoid of any detectable signal, confirming that the new *Slc6a5* mutation is a severe hypomorph or null allele (Fig. 2.F). This allele has been formally designated *Slc6a5^m1J^* by the Mouse Genome Informatics group at The Jackson Laboratory.

### Disinhibition of the spinal cord results in hastened neuromuscular junction maturation

*Slc6a5* is abundantly expressed in the ventral spinal cord and is required for the re-uptake of glycine and refilling of synaptic vesicles at inhibitory synapses [20]. Therefore, in the absence of GlyT2, sustained glycinergic inhibitory transmission is impaired. Motoneurons are normally under strong inhibition (reviewed in [2], part of which provides the negative feed-back necessary to end the volleys of action potentials that trigger muscle contractions. Therefore, in the absence of glycinergic inputs, motoneurons are more excitable and are presumed to fire excessively and without coordination, resulting in severe muscle spasms. The muscle tetanus phenotype observed in the *Slc6a5* mutants is consistent with such an overactivity of motoneurons (Movie S1), although we were unable to monitor motor neuron activity *in vivo* in response to either spontaneous or voluntary activation in unanesthetized mice. Nonetheless, this mutation allows the study of the role of increased motoneuron excitability on the maturation or maintenance of the neuromuscular system. We investigated the effects of this overactivity on two postnatal developmental features of the NMJ that had not been previously examined in the *Slc6a5* knockout mice: synapse elimination, and the molecular switch of the acetylcholine receptor subunit expression from the embryonic gamma subunit to the adult epsilon subunit.

In rodents, NMJs are innervated by more than one motor axon terminal at birth. During the first two postnatal weeks, pruning of extranumerary axonal branches eventually results in neuromuscular junctions with their adult form, a single terminal from a motoneuron connecting to a single postsynaptic site on the muscle fiber [42]. This process of synapse elimination has been shown to be hastened by electrical stimulation of the motor nerves [43] but few *in vivo*, non-invasive alternatives exist to endogenously modify the activity of the motoneurons [44].

**Figure 3:**
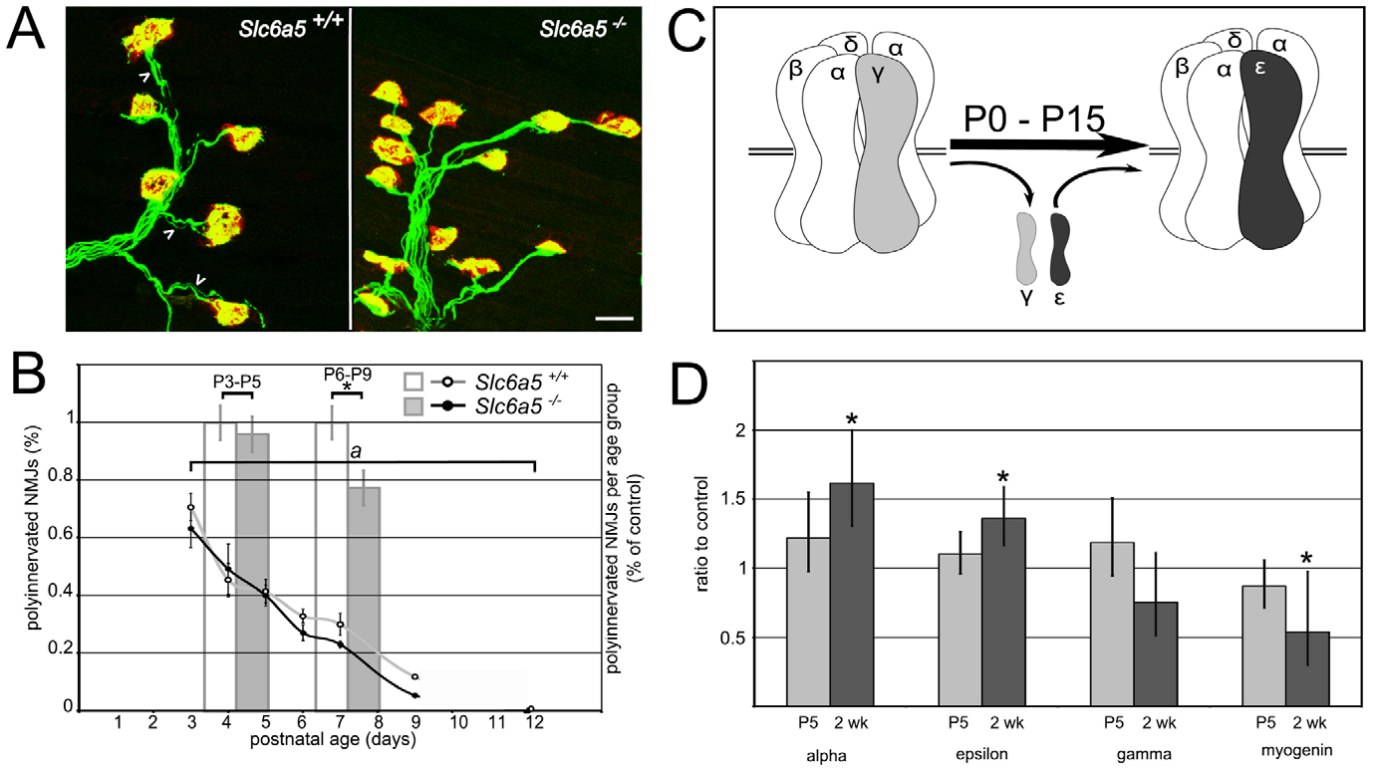
Neuromuscular junction phenotypes. **A.** Representative pictures of P7 NMJs in WT (left) and mutant (right) triangularis sterni muscles. The axons are labeled by the Thy1-YFP16 transgene (green) and the acetylcholine receptor plaques by fluorescent alpha-bungarotoxin (red). NMJs innervated by more than one axon terminal (arrowheads) are more abundant in the WT than in the mutant. **B.** Time course of the synapse elimination**.** The curves represent the average percentage of polyinnervated NMJs in wild-type (gray) and mutant (black) triangularis sterni muscles at ages P3 to P12. a: Wilcoxon signed-rank paired test, p = 0.09. The bars represent the average percentage of polyinnervated NMJs in the P3-P5 and P6-P9 age groups. * : Mann-Whitney test, p<0.05. Error bars represent the SEM. **C.** Schematic representation of the molecular switch from gamma to epsilon AChR subunits in the muscle pentameric acetylcholine receptor, also indicating the constitutive subunits alpha, beta and delta. **D.** Quantification of the fold-change of AChR subunits alpha, gamma and epsilon, and myogenin, in P5 and two week old synaptic regions of the mutant diaphragm, expressed as a ratio to age-matched WT values ( = 1.0). Biological replicates: 5 for P5, 7 for two week. Error bars represent the confidence interval. * : Mann-Whitney test, p<0.05.

Therefore, we quantified synapse elimination in the triangularis sterni muscle (Fig. 3.A), a flat muscle from the inner wall of the rib cage where NMJs can be clearly visualized. At P3, about 75% of the NMJs were polyinnervated, with the same number of extranumerary terminals per NMJs in both wild-type and mutant animals (50% of NMJs receiving 2 axon terminals, 20% of NMJs, 3 terminals and 5% of NMJs, 4 terminals, data not shown), and the elimination of all but one axonal branch was completed by P12 for both the mutants and wild-type (Fig. 3.B). However, the course of the elimination in the mutant mice significantly preceded that of their wild-type littermates after P5. The NMJs themselves were indistinguishable in size and AChR labeling intensity at P6-7, but a trend towards smaller NMJs with stronger labeling was evident in P9-11 samples (area: WT 166.6±10.2 µm^2^, mut 141.4±10.4, T-test p = 0.16, labeling intensity 154.6+/−5.5 arbitrary units, 171.6+/−7.9, p = 0.13 in samples from 4 mice of each genotype at each age).

Acetylcholine receptor expression is indeed another aspect of neuromuscular junction maturation, particularly the developmental switch from the gamma acetylcholine receptor subunit to the epsilon subunit (Fig. 3.C) [45]–[47]. This molecular switch results in the change of the AChR conductance required for the maintenance of the NMJs postnatally [48]. Experiments in vitro suggested that activity might regulate this developmental switch [49] and surgical denervation in mouse has been shown to prevent the increase in epsilon in newborn mice [50]. However, the effects of the reciprocal increase in axonal activity are less well studied *in vivo*.

We used syber-green QPCR to quantify the relative abundance of different AChR subunits in wild-type and mutant mice, at P5, an age when the switch from gamma to epsilon is actively taking place, and at two weeks, when epsilon has completely replaced gamma in wild type mice. No significant change in the AChR subunit expression was detected at P5. At 2 weeks, alpha and epsilon AChR subunits were expressed at higher level in the mutant muscle than in the control (Fig. 3.D). Alpha mRNAs were increased by almost 60%, and epsilon mRNA by 30%. Gamma mRNAs tended to be decreased, but no statistical significance was obtained with the sample size used. In wild type mice, the epsilon mRNA expression increases from birth to P15 before decreasing to its adult levels, whereas gamma mRNA decreases from birth to P15 [50]. The overexpression of epsilon and the slight reduction of gamma in the *Slc6a5* mutants are consistent with a hastened maturation of the NMJs. However, the elevated levels of alpha mRNAs in the *Slc6a5* mutants also suggest that the increased excitability of motoneurons drives a non-specific increase of postsynaptic transcription of the AChR subunits. Myogenin, among other myogenic factors, positively regulates the transcription of alpha and gamma AChR subunits [51]–[53] but is negatively regulated by innervation and electrical activity [54]–[57]. The increase in AChR subunit transcription is not consistent with the expected decrease of myogenin resulting from an increased activity. We therefore also measured the variations in myogenin RNA levels and found that at P5 and two weeks, myogenin levels were decreased by 25% and 50%, respectively, at these ages in the *Slc6a5* mutants (Fig. 3.D). This is consistent with an increase in motoneuron activity, indicating that hyperactivity of motoneurons *in vivo* positively regulates AChR transcription in a myogenin-independent way.

### No change in pain sensitivity in *Slc6a5* heterozygotes

**Figure 4:**
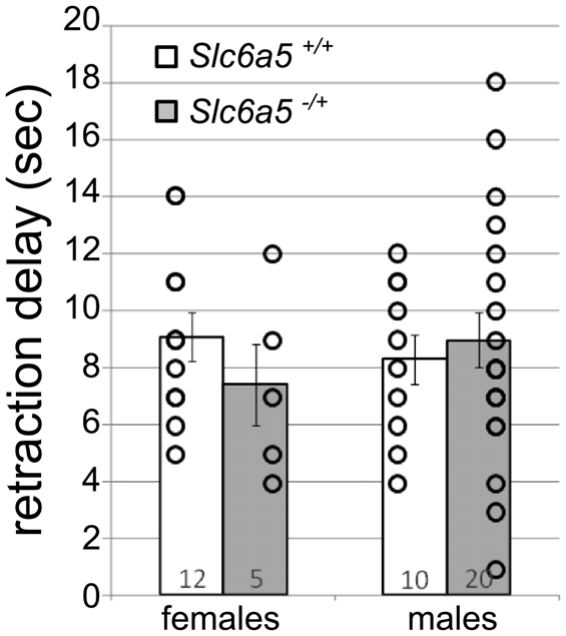
Normal thermal nociception in heterozygotes. Results of the hot-plate assay. Reaction time before flickering/retraction of one paw after mice have been placed on a 50 deg hot plate. Individual values are shown by circles, mean +/- SEM are shown by the chart and error bars. The number of mice tested is indicated.

Western blot analysis revealed that the levels of transporter are reduced by half in mice heterozygous for the *Slc6a5* mutation (Fig. 2.E). The consequences of this reduction are untested, but may differ from the complete loss of function in the homozygotes. A reduction in GlyT2 may diminish glycine re-uptake from the synaptic cleft, without hampering the neurotransmission itself. This would result in a longer half-life of glycine in the extracellular space, and an increased inhibition of the postsynaptic neurons through the increase of hyperpolarizing currents mediated by the glycine receptors. *Slc6a5* heterozygotes, therefore, could mimic the partial blockade of GlyT2 achieved pharmacologically. The specific GlyT2 blocker ALX1393 was recently shown to have antinociceptive effects in the rat [58]. We therefore compared thermal nociception between mice heterozygous for the *Slc6a5* mutation and wild-type littermates using a hotplate assay to determine if *Slc6a5* heterozygotes had a phenotype consistent with pharmacologically reduced GlyT2 activity. The delay in paw retraction in response to heat was not significantly different in the heterozygous mice (Fig. 4). In older mice, a slight but non-significant increase in the retraction time was noticed (data not shown). Thus, different effects on thermal nociception are obtained in *Slc6a5* heterozygous mice and ALX1393 treated rats.

### Partial loss of GlyT2 increases grooming behavior and context-dependent locomotion, but does not affect anxiety levels

*Slc6a5* is expressed at lower levels in the brain than in the spinal cord. In addition to the brain stem, it has been detected in the cerebellum, thalamus, the basal ganglia and the hippocampus by immunocytochemistry [13]. Transgenic mice expressing a fluorescent protein under the control of the *Slc6a5* gene also provided evidence for innervation of the amygdala and neocortex by a few *Slc6a5*-positive fibers [41]. Given the reduced amount of transporter in *Slc6a5* heterozygotes, and the possibility that GlyT2, by regulating extracellular glycine concentrations could modulate NMDA receptors activation, we tested other behavioral effects of reduced GlyT2. In particular, we wanted to examine phenotypes relevant to schizophrenia and anxiety given the association between *SLC6A5* and NMDA receptors and these conditions in humans.

**Figure 5:**
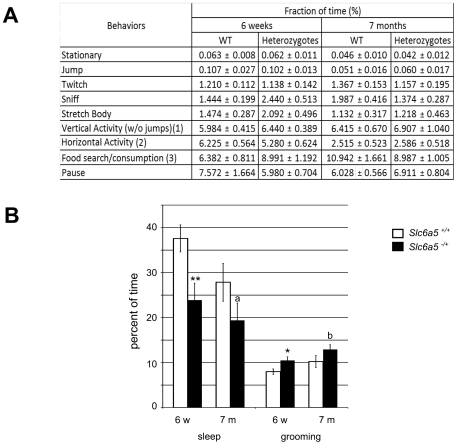
Nocturnal behavioral phenotypes in the home cage monitoring system. **A.** Table of the major behaviors that did not show any change between genotypes, in % of the total nocturnal time (mean and SEM). **B.** Behaviors with a significant change. *a* : p = 0.08 for the amount of sleep and b : p = 0.06 for the amount of grooming in the 7 months old mutants, * : p<0.05, ** : p<0.01, Mann-Whitney test. See “Methods” section for more detailed description. 6 female mice per age group and genotype were observed.

Heterozygous and wild-type mice at six weeks and seven months of age were used for these studies. In a first approach to detect spontaneous, non-induced (context-independent) differences in behaviors, mice housed individually were monitored over-night in their cage. Videos were then analyzed using software able to recognize and tabulate a large number of behaviors [59]–[61]. Many of the most frequent behaviors, such as horizontal locomotion, hanging, searching, or consuming food were not significantly different between the two genotypes (Fig. 5.A). However, heterozygotes spent less time sleeping during the dark period, their normal nocturnal activity phase, and more time grooming (Fig. 5.B). When considered by itself, independently of the other feeding behaviors, chewing was also more frequent in the young heterozygotes (data not shown).

**Figure 6:**
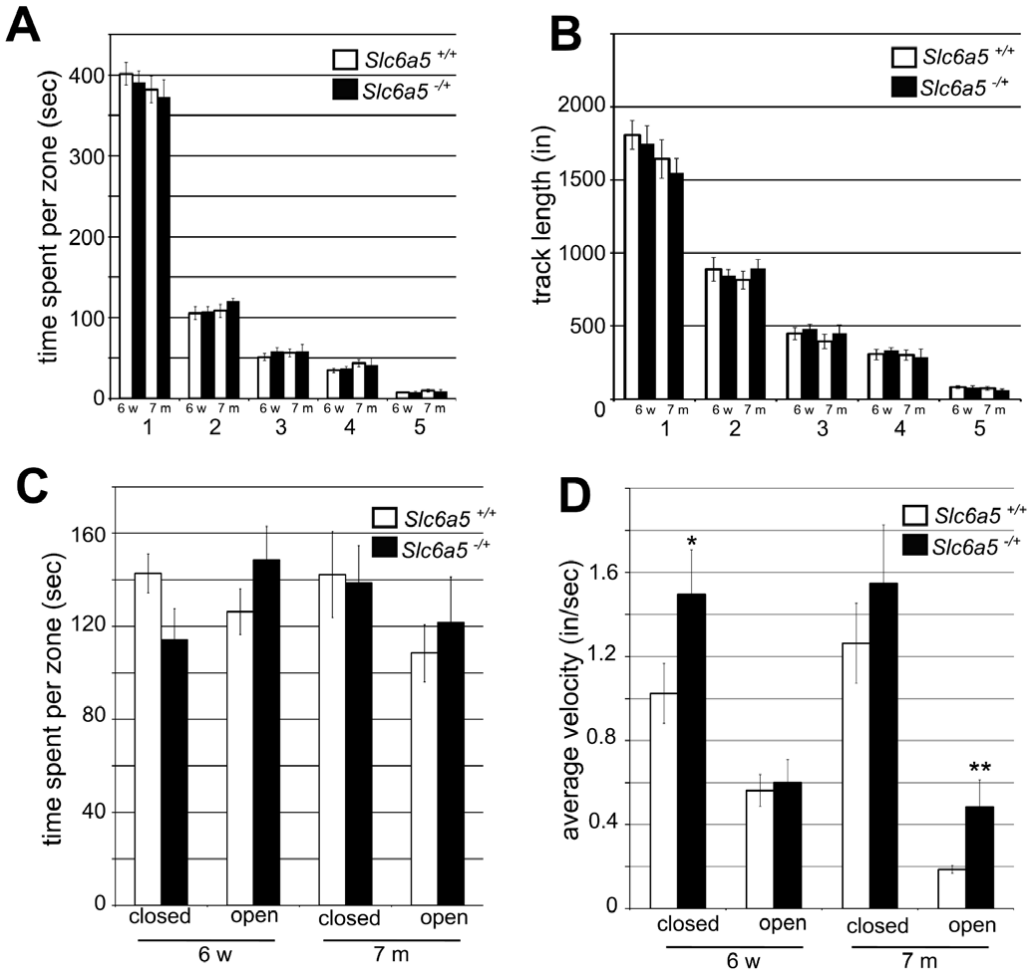
Anxiety-related behaviors. **A, B : Openfield test, C, D : elevated plus-maze test.** Wild-type and heterozygotes values are shown for two age groups, 6 weeks and 7 months old. **A.** Average time spent per zone of the openfield, in sec., zone 1 being the most peripheral, zone 5 the central zone. **B.** Average track length per zone (in inches) during the 10 min observation. **C.** Average time spent in the open or closed arm of the elevated plus-maze. **D.** Average velocity in the open and closed arms. 6 mice per age group and genotype were observed. * : p<0.05, ** : p<0.01, Mann-Whitney test.

To study anxiety-related behaviors and activity, we used openfield and elevated-plus-maze assays. In the openfield, the arena is virtually divided into 5 concentric zones. Mice spend more time in the most peripheral area (zone 1) than in the central area (zone 5), an indicator of anxiety consistent with the known apprehension of rodents to venture in open areas [62]. However, there were no differences in the time spent (Fig. 6.A) or the track length (Fig. 6.B) in each zone between the heterozygous and the littermate control mice at either age, suggesting that a partial decrease of *Slc6a5* is not anxiogenic. The elevated plus-maze has two opposing arms that are open and two opposing arms that are enclosed. Anxiety in this test is reflected by an increase in the time spent in the closed arms. Consistent with the open field results, there was no difference in the time spent in the different arms between the two genotypes (Fig. 6.C), indicating a normal anxiety level in the heterozygotes. Surprisingly, heterozygotes displayed an increased velocity that was significantly higher in the closed arm for the 6 weeks old, and in the open arm for the 7 months old group. This hyperactivity seems context-dependent since the reduction in sleep time observed in the home cage monitoring was not associated with a significant increase of locomotor behaviors, and the openfield did not show an increase in track length, which would indicate increased locomotor activity.

## Discussion

We have identified a new mutation in the mouse *Slc6A5* gene. This mutation arose on an NOD genetic background, but nonetheless shows many phenotypic similarities to previously reported knockout animals. We were able to use these mice to examine activity-dependent developmental processes at the postnatal NMJ, and to explore the behavioral impacts of reduced GlyT2 levels in viable heterozygous mice.

From a mouse genetics perspective, this mutation independently confirms the severe phenotype of *Slc6a5* mutations. Like the knockout, this allele also appears to be a null, based on undetectable protein levels. Importantly, the NOD background does not appear to markedly alter the severity of the phenotype. Also, the mutation is the result of a MusD retroviral insertion into intron 5 of the gene, resulting in the inclusion of a 183 bp insert in the *Slc6a5* mRNA, but resulting in a complete loss of protein. The MusD insertion is an interesting mechanism for a spontaneous mutation and has only been reported in one previous case that we are aware of [63]. MusD elements are closely related to ETn elements, which are a common cause of insertional mutations in mice. MusDs encode the proteins necessary for transposition, whereas ETns do not. Thus MusD encoded proteins cause ETns to mobilize, but the MusDs rarely mobilize themselves [64]. Our sequencing is consistent with the intron 5 insertion being a MusD1 element, although the shorter size (6 kb versus 7 kb) raises the possibility of an internal deletion of the retrotransposon in *Slc6a5^m1J^*.

The changes observed at the NMJ, including a hastening of synapse elimination and a decrease in myogenin expression, are anticipated for a mutation that would increase the firing activity of motor neurons based on impaired glycinergic transmission in the spinal cord, although we have been unable to directly record an increase in motor neuron firing over time in unanesthetized animals. The overt phenotypes of tetany in response to stimulation and rigidity due to increased muscle tone are also consistent with such a change in the spinal cord. Interestingly, both the NMJ phenotypes and the overt spastic phenotype are only visible after P5, suggesting that the lack of glycinergic inhibition only affects the excitability of motoneurons past a certain maturation stage. Elevation in transcription of multiple AChR subunits including both constitutive (alpha) and adult (epsilon) forms is surprising, particularly in light of the decrease in myogenin expression. This finding suggests an activity-dependent, myogenin-independent mechanism that regulates AChR expression.

In other model organisms such as Drosophila, mutation that effect excitability of the nervous system, such as Potassium channel mutations, have been extensively used to study the contribution of neuronal activity to developmental processes [65]–[67]. Our results suggest that *Slc6a5* mutations may be similarly useful for exploring these relationships, particularly in the mammalian peripheral nervous system and spinal cord.

The behavioral changes in heterozygous mice also provide a valuable avenue for additional research. Glycine is increasingly appreciated as an important neurotransmitter system in the brain as well as the spinal cord, and variation in genes affecting glycinergic transmission are associated with a variety of human conditions. Glycine has two potential modes of action in the brain. First, it can produce inhibitory chloride currents through activation of classical pentameric glycine receptors. Second, it can potentiate NMDA receptor activation through synaptic spill over. We noted a normal response to thermal nociception, decreased nocturnal sleep and increased grooming, and increased context-dependent activity in a T-maze, but no anxiety phenotypes in *Slc6a5* heterozygous mice. The normal nociception suggests that *Slc6a5* heterozygotes do not have glycine reuptake defects as severe as rats treated with GlyT2 inhibitors. The analgesic effect of GlyT2 blockers in rats is consistent with the observation that pharmacological blockade of GlyT2 in spinal cord sections increases the decay time of evoked and spontaneous inhibitory currents, i.e. increases extracellular glycine [68]. Although insufficient to effect pain sensitivity, a slight increase in extracellular glycine could have other consequences in the brain, through the glycine-dependent NMDA receptor potentiation. For instance, decreased glycine transport has been shown to increase NMDA receptor-mediated currents [68]. Effects of changing GlyT2 activity had not been tested in the brain previously, and our behavioral phenotypes such as decreased sleep, hyperactivity and repetitive behaviors were, to our surprise, not consistent with increased glycinergic inhibition or NMDA receptor potentiation. Hyperactivity and stereotypy have been observed in mice with reduced amounts of NMDA receptors [69], or reduced NMDA receptor function, caused either by mutation of its glycine binding site [70] or reduction of the synthesis of another endogenous ligand of the glycine-binding site, D-serine [71]. These mice are recognized models of schizophrenia, respond to schizophrenia drug therapies and support the theory of NMDA receptor hypofunction of schizophrenia (reviewed in [72] and [73]). The decrease in nocturnal sleep we observed in the *Slc6a5* heterozygotes is also reminiscent of the sleep disorders commonly associated with schizophrenia [74]. Therefore, while mice heterozygous for mutations in *Slc6a9* show phenotypes consistent with reduced glycine transport, such as increased glycinergic facilitation of NMDA receptors and anti-schizophrenic effects [34], [35], the mice heterozygous for the mutation in *Slc6a5* have behavioral phenotypes that could be explained by a reduced glycine secretion and a decreased glycinergic facilitation of NMDA receptors in the brain. In support of this interpretation, pharmacological GlyT2 blockade has been shown to not only increase extracellular glycine concentration, but also have a presynaptic effect that could be explained by a reduced cytosolic glycine concentration limiting the synaptic vesicle release probability [68]. Our results, together, suggest that reduced GlyT2 activity may have different effects in the brain versus the spinal cord, or there may be a greater contribution of NMDA receptor potentiation in one region versus the other. However, the similar spectrum of behavioral changes compared to other schizophrenia mouse models is intriguing considering the association of a SNP near human *SLC6A5* with schizophrenia [37].

In summary, the *Slc6a5* mutant mice provide a useful model for examining activity-dependent developmental processes and in that context, suggest the presence of an activity-dependent, myogenin-independent mechanism for regulating AChR expression in muscle. The behavioral changes observed in heterozygotes are also indicative of a function for *Slc6a5* in behaviors controlled by higher brain regions. The affected circuits underlying these changes can now be explored both anatomically and pharmacologically based on *Slc6a5* expression and this functional demonstration of its relevance.

## Materials and Methods

### Animal studies

All studies were performed in accordance with the Guide for the Care and Use of Laboratory Animals, and all procedures were approved by The Jackson Laboratory Animal Care and Use Committee, comprehensive protocol #01026, approval date Oct. 28, 2010. Strains, ages, and the number of animals examined in given experiments are provided in the text and figure legends at appropriate points. All experiments, except the mapping of the mutation and the synapse elimination count (described below), were performed with mice from the original NOD CD8 colony. Animals were provided food (NIH 6% diet) and water ad libitum and were kept on a standard 14-10 light-dark cycle.

### Mapping of the new mutation

Ovaries of affected, P10 females from the NOD CD8 colony (NOD.CgPrkdc^scid^Emv30^b^/Dvs) were transplanted in NOD SCID recipient females. Recipient females were mated with DBA/2J males and the F1 obligate heterozygotes were mated together. Affected and non-affected mice of the F2 generation were collected. When it appeared that the mutation partially segregated with the white coat color linked to the tyrosinase gene, at 94.6 Mb on chromosome 7, we searched the Mouse Genome Database (Blake et al., 2011) (http://www.informatics.jax.org) for existing mutations with neurological phenotypes that would already have been reported on chromosome 7 between 30 and 150 Mb. The phenotype of the knock-out of *Slc6a5* closely resembled the new mutation; therefore, we selected MIT SSLP markers around this locus that are polymorphic between NOD and DBA/2 (see Fig. 1.D). Genotyping 16 affected and 19 unaffected mice of the F2 progeny with these markers confirmed that the mutation segregated with D7MIT27 at 51.2 Mb, 6 Mb away from *Slc6a5*. A separate colony on the original inbred NOD mice was also maintained for use in behavioral studies.

### Primer Sequences for the characterization of the mutation

The primers used for the sequencing of *Slc6a5* mRNA were ex1F : TTT GAT TGG TTT TAC AGT GAA GTA A and ex2R : CTC GTC CTC CGG TAT GGT AG, amplifying a 689 bp product ; ex2F : GTG GCC ACC ACT ACC ATA CC and ex7R : AAT CAC CCA AGC CAA GAA AA, amplifying a 699 bp product ; ex7F : CTT TTC TTG GCT TGG GTG AT and ex13R : GGT GTC CAC AAG CTG AAA CA, amplifying a 705 bp product, and ex13F : TGA TCA CAC AGG GTG GAA TTT and ex16R : GAG GAA GCC CGG GAG TAA TA, amplifying a 578 bp product. The insertion site of the MusD retrotransposon was determined by PCR on genomic DNA using int5F : AGA GCA CTG AAG AGG CAA GC and MusR : GAA CGG TTC GAC TGA GAA GG. For the amplification of the retrotransposon by primers in intron 5 flanking the insertion site we used int5F and int5R : GGG AAC TTC CTT TCC AGT CAG. The following primers were used to genotype for the mutation from genomic DNA: *Slc6a5* F (in intron 5, 5′ of the insertion) : TGC TCT CTT TTG GTC TTA TTC AAA, *Slc6a5* WT R (in intron 5, 3′ of the insertion) : CCA GTA GGT GGT GCT GTT GG and *Slc6a5* Mut R (in the retrotransposon) : TAG ACG GGG CAA AAG AAG AA.

### Immunocytochemistry

Reagents used consist of a guinea-pig anti-GLYT2 antibody (Millipore, AB1773) and the Vectastain ABC Kit for Guinea Pig IgGs (Vector Laboratories, PK-4007). After intracardiac perfusion with PBS followed by 2% paraformaldehyde in PBS, spinal cords and brains were harvested and post-fixed overnight in 2% paraformaldehyde in PBS at 4 degrees. Tissues were dehydrated and paraffin-embedded. 4 um-thick sections were de-paraffinized and rehydrated in a xylene/ethanol series. An antigen-retrieval step was required, consisting of heating the slides in citrate buffer (10 mM, pH 6) in a microwave twice to a boiling point. Endogenous peroxidases were blocked with Peroxo-Block (Invitrogen, 00-2015) for 3 min. Sections were incubated overnight at 4 degrees in the primary antibody diluted in 0.5% BSA in PBS, then at room temperature in a biotinylated goat anti-guinea pig antibody for 30 min, in the avidin/HRP conjugate for 45 min and stained with DAB for 5 min. Sections were rinsed, nuclei were counterstained in hematoxylin for 3 min, dehydrated and mounted in Permount medium (Fisher Scientific).

### Western blotting

Spinal cords were collected and homogenized in 250 mM sucrose, 50 mM Tris pH 7.4, 5 mM MgCl2, 1 mM DTT and protease inhibitors (Complete protease inhibitor cocktail, Roche), at 4 degrees. Lysates were centrifuged at 6.000 g for 15 min and the supernatant was ultracentrifuged at 100.000 g for one hour. The pellet was solubilized in 20 mM Tris pH 7.4, 0.4 M NaCl, 15% glycerol, 1 mM DTT, 1.5% Triton X100. 50 µg of proteins (as measured by the BCA method (BCA Protein Assay, Pierce) were heated at 70 deg for 5 min in reducing Laemmli sample buffer and used for the western-blot following usual protocols. Membranes were probed with a guinea pig anti-GLYT2 primary antibody and an HRP-conjugate anti-guinea pig secondary antibody (Millipore, AP108P). For a loading control, membranes were stripped in 25 mM glycine (pH 2) for one hour and re-probed with a mouse anti-beta-dystroglycan primary antibody (MANDAG2, clone 7D110) and an HRP-conjugate anti-mouse secondary antibody (Perkin Elmer).

The monoclonal antibody MANDAG2 developed by G.E. Morris was obtained from the Developmental Studies Hybridoma Bank developed under the auspices of the NICHD and maintained by The University of Iowa, Department of Biology, Iowa City, IA 52242.

### NMJ visualization

To visualize NMJs, *Slc6a5* mutants were bred to Thy1-YFP16, a transgenic line expressing YFP in all motor neurons from embryonic development onward [75]. Ribcages were fixed overnight in 2% paraformaldehyde in PBS at 4 degrees, the triangularis sterni was dissected out and stained in Alexa-594-conjugated alpha-bungarotoxin (Invitrogen) to label AChR clusters. Quantification of the polyinnervation was performed blind of the genotypes on a Nikon microscope, with a 63X objective. NMJs were called monoinnervated when only one axon terminal was seen in apposition with the AChR plaque, and polyinnervated when 2 or more axon terminals where seen. An average of 50 NMJs per mouse and 8 mice per age group and genotype were examined.

### Quantitative PCR

Diaphragms were collected and stored overnight in RNALater (Applied Biosystems, AM7021). Endplates, visualized as the central part of the muscle containing the innervating nerves, were dissected. RNAs were extracted with the TRIzol protocol (Invitrogen, 15596-026) and assessed for purity on a Nanodrop spectrophotometer (ThermoScientific). Only samples with a 260/280 ratio above 1.9 were used. 500 ng of total RNAs were reverse-transcribed with oligo dT priming following manufacturer instructions (SuperScript™ III Reverse Transcriptase, Invitrogen, 18080-093) and 50 or 10 ng of cDNA were used per quantitative PCR reaction. qPCR reactions were run with the SYBR-Green PCR master mix (Applied Biosystem) on a 7500 Real-Time PCR System (Applied Biosystems).

Primers were selected from PrimerBank [76], for GAPDH (PrimerBank ID 6679937a1, AGG TCG GTG TGA ACG GAT TTG and TGT AGA CCA TGT AGT TGA GGT CA), myogenin (PrimerBank ID 13654247a1, GAG ACA TCC CCC TAT TTC TAC CA and GCT CAG TCC GCT CAT AGC C) and the AChR subunits alpha, epsilon and gamma (resp. PrimerBank ID 31542391a1, CTC TCG ACT GTT CTC CTG CTG and GTA GAC CCA CGG TGA CTT GTA ; PrimerBank ID 6752950a3, GGC AGC TTT TAC CGA GAA TGG and CGG CGG ATG ATG AGC GTA TAG ; PrimerBank ID 31982815a3, CCC GAC GGT TGT ATC TAC TGG and CTG GGA TTG GAA GAT GAG GGA). We verified that all primers presented the same amplification efficiency for a range of 1 to 100 ng of cDNA per PCR reaction and used the ΔΔCt method to represent the fold change of each target mRNA in the mutant samples compared to the wild-type one. Error bars represent the extreme values of the fold change incorporating the standard deviations of the mutant ΔCts. Muscles were harvested from mice at P5 (n = 5) or at P12 and P16 (4 and 3 samples resp.), which showed no differences and were pooled and reported as “two weeks” samples.

### Hot plate

3 to 5 month old naïve mice were tested only once on a dry bath incubator (Fisher Scientific) plate set at 50 degrees. We measured the response latency until the first hind paw flick was observed. The experiment was stopped after the first response or after 30 sec on the plate if no response was observed in this time frame.

### Home Cage Monitoring

We used the Home Cage Scan system from CleverSys [59]–[61] consisting of 4 cameras monitoring simultaneously 4 mice individually housed in 4 separate 13×18×29 cm acrylic cages on a 12:12 light:dark cycle. The room was lit by white lights from 6 am to 6 pm, and red lights from 6 pm to 6 am. Mice were introduced in the monitoring cages one hour before dark. Recordings were made from 1 hour before dark to 1 hour after dark and analysis of the recorded behavior was made on the 12 hours of the dark cycle. Random bouts of the movies were inspected after the experiment to ascertain the accuracy of the behaviors called by the recognition software. 6 female mice per genotype and age group in the defined NOD genetic background were monitored for 2 successive nights. Results are presented as the average percent of time where a given behavior was observed. The behaviors presented in this article did not show any significant variation between the first and the second night. In Fig. 5.A, “vertical activity” (1) regroups the behaviors Come Down, Come Down From Partially Reared, Come Down To Partially Reared, Hang Cuddled, Hang Vertically From Hang Cuddled, Hang Vertically From Rear Up, Land Vertically, Rear Up, Rear up From Partially Reared, Rear up To Partially Reared, Remain Hang Cuddled, and Remain Hang Vertically; “horizontal activity” (2) regroups the behaviors Turn, Walk Left, Walk Right, Walk Slowly; and “food search/consumption” (3) regroups the behaviors Chew, Dig, Eat (from the grain holder).

### Open Field

The openfield (BiObserve) consists of an open square area (24″×24 ″) divided in 5 concentric zones (each approximately 4.8 ″ wide) monitored by a camera placed above for 10 min per mouse. Movies were analyzed with the BiObserve software and the track length and time per zone were used for analysis.

### Elevated plus maze

The elevated plus maze (Columbus Instruments) consists of 4 arms (11.75” long x 2” wide), 2 of which are open and 2 closed (by 6” high walls) on a metal platform about 51” off the floor. Naïve mice were placed in the center of the 4 arms while number of entries per arm, time spent and track length in each arm, number of fecal boli and urination were recorded for a 5 minute period. Entries were recorded as number of photo-beam breaks. Time spent in each arm and track length were recorded with a CCD camera above the plus-maze coupled to the BiObserve analysis software, and all other parameters were measured manually. Average velocity was calculated by dividing the track length by the time spent in each arm.

### Statistics

Differences were considered significant for statistical tests returning p values <0.05 (*), <0.01 (**) and <0.001 (***).

For the analysis of the synapse elimination counts in Fig. 3.B, the Wilcoxon signed-rank test was applied on the average values of the mutant and control groups paired by age from P3 to P12. To take in better consideration the variations inside each age/genotype group, individual observations were pooled into a P3-P5 and a P6-P9 groups with the percent of polyinnervated NMJs expressed as a percent of the average in the control group at each age, and analyzed with the Mann-Whitney test. The age groups were determined based on the observations that the overt spastic phenotype is not visible before P5 in the mutants and that there seem to be no difference in the time course of the synapse elimination before P5, but a difference after P5.

Statistical significance for the quantitative PCR results in Fig. 3.D was tested by comparing the ΔCts to GAPDH for each target in the control and mutant groups with the Mann-Whitney test. The Mann-Whitney test was used to compare the times spent performing the indicated behaviors (Fig. 5.B) and the average velocities in the elevated plus maze (Fig. 6.D).

## Supporting Information

Movie S1 The handling induced spasticity of a *Slc6a5* mutant pup from the original mapping cross. Note the affected mouse is albino. In response to stimulation, the mouse becomes rigid and spastic, consistent with overactivation of motor neurons in the absence of glycinergic inhibition in the spinal cord.
(WMV)
